# Enhancing the performance of an in vitro RNA biosensor through iterative design of experiments

**DOI:** 10.1002/btpr.70005

**Published:** 2025-03-12

**Authors:** Rochelle Aw, Karen Polizzi

**Affiliations:** ^1^ Centre for Synthetic Biology, Imperial College London London UK; ^2^ Department of Chemical Engineering, Imperial College London London UK; ^3^ Present address: Department of Bioengineering Stanford University Stanford California USA

**Keywords:** biosensor, definitive screening design, mRNA integrity, mRNA vaccines

## Abstract

The quality control of RNA has become increasingly crucial with the rise of mRNA‐based vaccines and therapeutics. However, conventional methods such as LC–MS often require specialized equipment and expertise, limiting their applicability to high throughput experiments. Here, we optimize a previously characterized RNA integrity biosensor, that provides a simple colorimetric output, using Design of Experiments (DoE). Through iterative rounds of a Definitive Screening Design (DSD) and experimental validation, we systematically explored different assay conditions to enhance the biosensor's performance. Optimization led to a 4.1‐fold increase in dynamic range and reduced RNA concentration requirements by one‐third, significantly improving usability. Notable modifications included reducing the concentrations of reporter protein and poly‐dT oligonucleotide and increasing DTT concentration, suggesting a reducing environment for optimal functionality. Importantly, the optimized biosensor retained its ability to discriminate between capped and uncapped RNA even at lower RNA concentrations. Overall, our improved biosensor offers enhanced performance and reduced sample requirements, paving the way for rapid, cost‐effective RNA quality control in diverse settings, including resource‐limited environments.

## 1 INTRODUCTION

With the emergence of ground‐breaking technologies like mRNA vaccines and related therapeutics, the quality control of RNA has gained even greater significance.^1^ Unlike DNA, RNA is inherently less stable due to its single‐stranded structure and the presence of ribose sugars instead of deoxyribose sugars. The susceptibility of the hydroxyl group in RNA to hydrolysis further contributes to its increased likelihood of degradation.^2^ This inherent instability necessitated the initial ultra‐low temperature storage requirements for SARS‐CoV‐2 mRNA vaccines, posing challenges for distribution in rural areas.^3^ Consequently, there is an urgent need to ascertain the integrity of RNA not only during production, but also potentially at the point of use. Thus, it becomes crucial to develop tests that can be performed by non‐experts and without the need for specialized equipment. While RNA produced by in vitro transcription (IVT) is more stable than mRNA in vivo, partly due to the stability afforded by formulation, one of the main problems with IVT mRNA is the lack of natural capping machinery, which improves RNA stability, translation efficiency and immune recognition.^4^ To compensate for this, IVT mRNA is either capped post‐transcriptionally by enzymatic treatment or co‐transcriptionally by adding chemical cap analogs to the reaction.^5^

Various methods currently exist to assess RNA integrity reviewed in Ref. [6] including gel electrophoresis,^7^, ^8^ molecular mobility, template amplification,^9^, ^10^ complementary oligonucleotide hybridization,^11^ aptamer probes,^12^ fluorescent biosensors,^12^ liquid chromatography^13^ or protein‐RNA interactions.^14^ These techniques typically generate outputs based on fluorescence, light refraction/transmission, or electrical current, making them challenging to implement in non‐laboratory settings. Although biosensors have been developed to enhance diagnostic analysis for RNA integrity, they still possess certain limitations.^15^, ^16^ One of the difficulties with each of these techniques is that they require specialized equipment, and trained professionals to perform the experiments.

**FIGURE 1:**
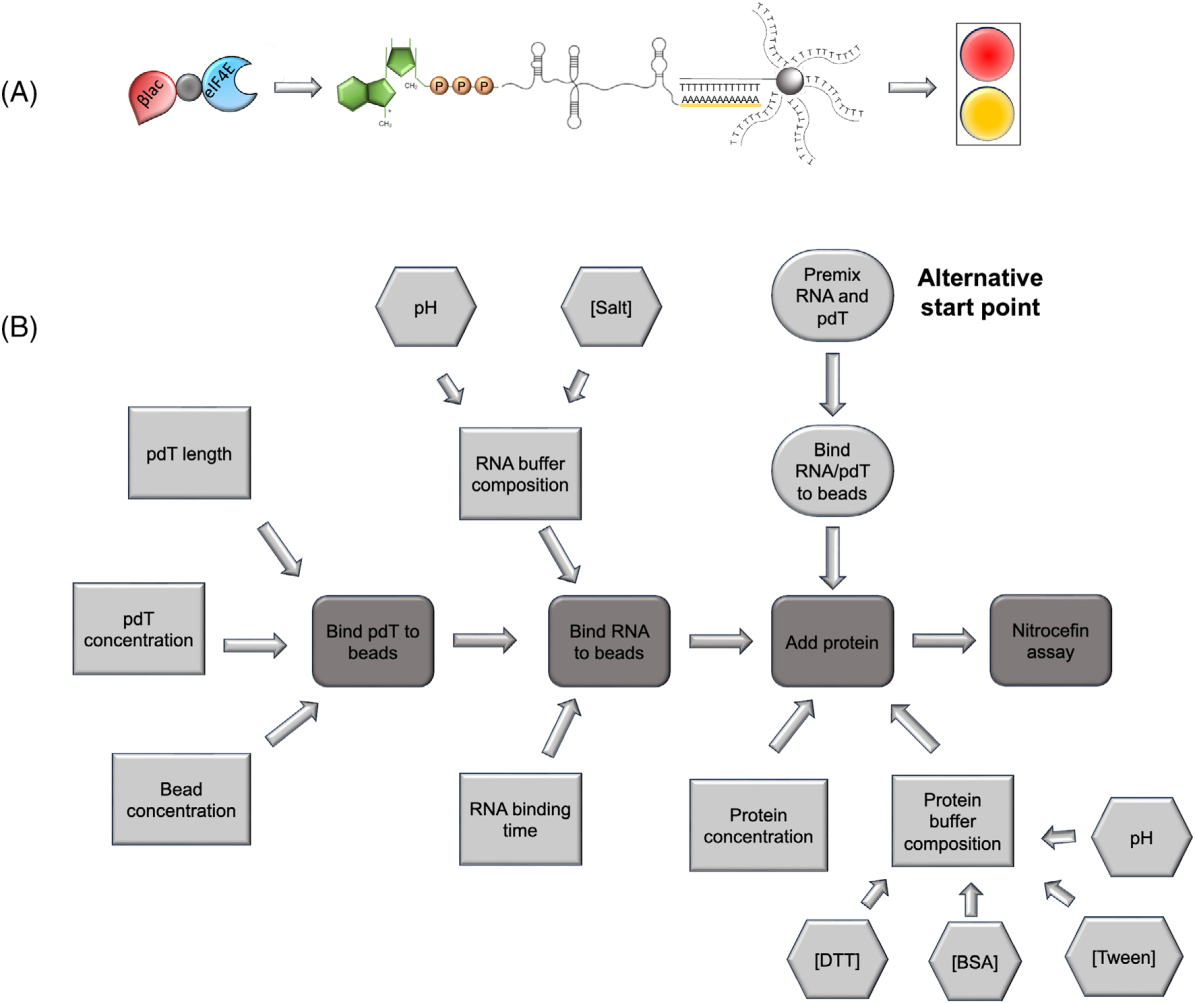
Experimental design for biosensor assay optimization. (a) Schematic of the biosensor assay workflow, with binding of the eIF4‐β‐lactamase complex to the 5′ cap of the RNA. Presence of a polyA tail allows binding to a poly‐dT biotinylated oligonucleotide bound to a streptavidin bead. Development with nitrocefin shows a color shift from yellow to red in the presence of full‐length RNA. (b) A process flow diagram of decision making for the design of experiment setup with different components being modified. Dark gray represents the workflow of the protocol, with the oval shapes indicating an alternative starting point for the assay. Rectangular boxes are conditions that can be altered at each step, and if multiple components factor into these changeable parameters these are indicated by hexagonal boxes.

Previously, we have developed a biosensor that is capable of recognizing the m7G cap structure and the polyA tail simultaneously to quantify the percentage of intact RNA in a sample.^17^ The biosensor uses an engineered chimeric protein (B4E, a fusion of murine eIF4E protein and a β‐lactamase) that recognizes the 5′ cap, and biotinylated deoxythymidine oligonucleotide‐functionalized beads that bind the polyA tail. In the absence of one or both of these two components, no signal is observed. The biosensor was also designed to result in a color change as a visualizable output, making this a low‐tech approach that does not need to be deployed in a laboratory‐based setting if desired. While this biosensor is not capable of determining total RNA yield, it is accurate at observing degradation. However, there were limitations in the length of RNA that was able to be accurately observed, with the signal decreasing for longer RNA molecules. Although this could be compensated with using increased concentration in the assay, it meant that more than one dose of RNA vaccine would be needed for each quality control assay. Here, to counteract this limitation, we undertook a screen of different conditions using Design of Experiments (DoE) to improve the biosensor limit of detection and signal‐to‐noise ratio with longer RNA molecules (Figure 1).

We opted to use a definitive screening design (DSD) to evaluate the essential components of the biosensor assay. Originally described by Jones and Nachtsheim,^18^ this three‐level factor design removes the bias of secondary effects while limiting the number of runs. We established a total of eight factors to be of importance, which, according to Jones and Nachtsheim, is over the threshold where using a DSD is sufficient to cover the same design space as a full factorial design. Definitive screening designs are often used to identify key factors that have the most impact on the system, which can then be explored via a full factorial screen. However, it is possible to hone the design of the space using iterative DSD runs to move toward an optimum, this is beneficial if multiple components are identified as significant that would make a full‐factorial laborious and difficult to achieve.^19^ We used a stepwise model with a Bayesian information criterion (BIC) stopping point to fit the regression model for the DSD, which has been shown to be the most accurate method for out of sample predictions.^19^, ^20^, ^21^ This model analyzes the design using full quadratic analysis, giving indications of the main effectors in addition to two factor interactions.^22^ By pairing DSD with experimental validation over multiple rounds we have shown the importance of exploring the entire design space when designing and optimizing novel biosensors. Through DoE, we have created a biosensor that has a 4.1‐fold increase in dynamic range compared to our original design and that uses a third less RNA. Both changes increase the usability of the biosensor in general, but also in a non‐laboratory setting by increasing the signal of the biosensor, making changes more visible to the eye.

## 2 MATERIALS AND METHODS

### 2.1 Chemicals and media

Bacterial strains were cultured in Lennox lysogeny broth (LB) medium (1% peptone from casein, 0.5% yeast extract, 0.5% NaCl) supplemented with either 37.5 μg mL^−1^ kanamycin or 100 μg mL^−1^ ampicillin. Common chemicals including β‐D‐1‐thiogalactopyranoside (IPTG), dithiothreitol (DTT), bovine serum albumin (BSA), and phenylmethylsulfonyl fluoride (PMSF) were purchased from Sigma Aldrich (Dorset, UK). Tween‐20, Dynabeads™ MyOne™ Streptavidin T1, and Oxoid™ nitrocefin were purchased from ThermoFisher Scientific (Paisley, UK). Oligonucleotides were synthesized by Invitrogen (Paisley, UK). Restriction enzymes were purchased from New England Biolabs (Hertfordshire UK).

### 2.2 Strains and plasmids

Bacterial recombinant DNA manipulation was carried out in *Escherichia coli* strain NEB 5‐α (New England Biolabs). Protein expression was carried out in BL21 (DE3, New England Biolabs). The pET28a‐B4E (Addgene 162,067) and pRSET‐T3 plasmids were previously described.^17^ DNA encoding the SARS‐CoV2 spike protein and receptor binding domain (RBD) sequences was synthesized by GeneArt™ Gene Synthesis (ThermoFisher Scientific). The CFPS‐Spike and CFPS‐RBD plasmids were created using the Gibson DNA assembly method^23^ using the CFPS‐lucA plasmid^24^ as a backbone and replacing the luciferase insert with the Spike protein and RBD sequences, respectively. All plasmids were sequence verified (Eurofins Genomics, Ebersberg, Germany). Plasmids were extracted using the Qiagen mini prep kit (Manchester, UK) and linear fragments were purified using the Zymo clean and concentrate kit (Zymo Research, California, USA).

### 2.3 In vitro mRNA production

For in vitro transcription of both capped and uncapped RNA, the pRSET‐T3 was linearized with PspXI and the CFPS‐Spike or CFPS‐RBD plasmids were linearized with NruI. Capped mRNA was prepared using the HiScribe™ T7 ARCA kit (with tailing, New England Biolabs) according to the manufacturer's instructions. Briefly, reactions were carried out using 1 μg of linearised plasmid for 3 h at 37 °C before proceeding to the tailing reaction and DNase treatment steps.

To generate uncapped RNA, 1 μg of linearised DNA template was added to a 200 μL reaction containing 1× transcription buffer and 400 U of T7 RNA polymerase (ThermoFisher Scientific), 1.5 mM of each NTP (ThermoFisher Scientific) and 80 U murine RNase inhibitor (New England Biolabs). The reaction was incubated at 37 °C overnight. The DNA template was removed by digestion with 5 μL of DNaseI (New England Biolabs) for 1 h at 37 °C.

Both capped and uncapped RNA were purified using the RNA Clean & Concentrator‐25 (Zymo Research) to remove residual buffer and NTPs. RNA purity was checked by visualization on a bleach gel [8] and quantification was performed using a spectrophotometer.

### 2.4 RNA refolding

Prior to use in the biosensor assay RNA samples were refolded as previously described to restore the tertiary structure.^17^, ^25^ Briefly, after defrosting, the RNA was diluted to the concentration needed for the experiment in Buffer A (50 mM HEPES, 100 mM KCl, pH 7.4) or as specified by the design of experiments, and incubated at 80 °C for 2 min, followed by a further 2 min at 60 °C. MgCl_2_ was added to a final concentration of 1 mM and the sample was incubated for 30 min at 37 °C. Samples were stored on ice before use.

### 2.5 Protein purification

The pET28a‐B4E plasmid was transformed into BL21 (DE3) and grown overnight in LB containing 37.5 μg mL^−1^ kanamycin at 30 °C with shaking at 250 rpm. The following morning 1.25% (v/v) of the overnight culture was used to inoculate 200 mL of LB medium in a 2 L flask and grown at 25 °C until an OD_600_ of 0.5–0.6 was reached. Expression of B4E was induced by adding IPTG to a final concentration of 0.5 mM. The culture was left to grow for 16 h at 25 °C, 250 rpm. The culture was centrifuged at 4000 ×g for 20 min at 4 °C. The supernatant was removed, and the pellet resuspended in 5 mL of lysis buffer (50 mM NaH_2_PO_4_, 1 M NaCl, 20 mM imidazole, 0.1% Triton‐100X, 10 mM beta‐mercaptoethanol, 5% glycerol) per 1 g of wet cell weight, supplemented with 1 mM PMSF and 1 mg mL^−1^ lysozyme, and incubated at 4 °C for 30 min. The cells were disrupted by sonication for 140 s (10 s on, 10 s off, 35% amplitude) on ice. The soluble fraction was collected by centrifugation at 16,000 ×g for 30 min at 4 °C. The cleared supernatant was incubated with 1 mL of 50% Ni‐NTA agarose resin (Qiagen) per 4 mL of lysate for 1 h at 4 °C with gentle agitation. The sample and resin were loaded onto a polypropylene column and the lysis solution cleared through gravity flow. The column was washed twice with 4 mL of wash buffer (50 mM NaH_2_PO_4_, 1 M NaCl, 50 mM imidazole, 5% glycerol). Finally, a total of five elution fractions were collected in two steps: two fractions of 0.3 mL using elution buffer 1 (50 mM NaH_2_PO_4_, 1 M NaCl, 5% glycerol, 75 mM imidazole) and three fractions of 0.3 mL using elution buffer 2 (50 mM NaH_2_PO_4_, 1 M NaCl, 5% glycerol, 250 mM imidazole).

The fractions were checked for purity using an SDS‐PAGE gel and those with the highest concentration of protein were combined and buffer exchanged a total of three times into protein storage buffer (50 mM HEPES, 100 mM KCl, 10% (v/v) glycerol) using a 30 KDa MWCO Amicon® Ultra‐15 centrifugal column (Sigma Aldrich). The centrifugal column was used to concentrate the protein to 200 μL and the final concentration measured using the Pierce™ BCA protein assay kit (ThermoFisher Scientific).

### 2.6 Biosensor assay

The baseline biosensor assay was carried out as previously described^17^ with all modifications stated in the description of the experiment. All incubation steps were carried out at room temperature on a rotator at 60 rpm. Briefly, in PCR tubes, 10 μL Dynabeads™ MyOne™ Streptavidin T1 were washed three times with 20 μL Buffer A (50 mM HEPES, 100 mM KCl, pH 7.4) using a magnetic rack. The beads were then resuspended in 20 μL of 2× Buffer A (100 mM HEPES, 200 mM KCl, pH 7.4). 20 μL of 3.75 μM biotinylated poly‐dT (pdT25) in RNase free water were added and incubated for 10 min. The beads were washed with 20 μL of Buffer A, before 20 μL of refolded RNA sample at a concentration of 0.3 μM was added and incubated for 20 min. The beads were then washed with 100 μL of Buffer B (33 mM HEPES, 66 mM KCl, 0.1% BSA, 0.1% Tween‐20, 6 mM DTT, pH 7.4) before 50 μL of 0.45 μM B4E diluted in Buffer B was added and incubated for 1 h. The unbound protein was removed with three washes with 200 μL of Buffer B. The washed beads were resuspended in 200 μL of Buffer A of which 8 μL were used in the nitrocefin assay.

### 2.7 Nitrocefin assay

A total of 192 μL of 0.1 mM nitrocefin solution (in 50 mM phosphate buffer, pH 7) was mixed with 8 μL of beads in a 96‐well plate. The absorbance at 492 nm was monitored over time using a CLARIOstar plate reader (BMG Labtech Ltd., Aylesbury, UK). Readings were collected every 1 min for 90 min. All biosensor assays were run in triplicate and included a negative control replacing the capped RNA with uncapped RNA.

### 2.8 Design of experiments

A three‐level DSD was used to determine the importance of each of the components in the design of experiments using the JMP Pro 15 software (JMP UK & Western Europe, Marlow, UK). Nine factors were selected based on pre‐screening to determine the impact of the factor on the biosensor assay. The selected factors were the salt concentration and pH of the RNA buffer, the concentration of the pdT_25_ oligo, the concentration of the B4E protein, the concentrations of BSA, Tween‐20, and DTT in the protein buffer and the salt concentration and pH of the protein buffer (Figure 1). The upper and lower bounds were determined experimentally. Forward stepwise regression using a Bayesian information criterion (BIC) stopping point with a *p*‐value of 0.1 was performed using JMP to fit the regression model for the DSD. The data from the biosensor assay were transformed in one of three ways to use to fit the model: (i) end adjusted, where the final biosensor absorbance reading after 90 min was corrected by subtracting the negative control, (ii) end ratio, where the final biosensor absorbance reading after 90 min was divided by the negative control, and (iii) mid‐adjusted where the absorbance value after 10 min was corrected by subtracting the negative control. The DSD model was run a total of 4 times with each run including the data from all previous runs.

## 3 RESULTS AND DISCUSSION

### 3.1 Maximizing RNA capture to improve signal strength

**FIGURE 2:**
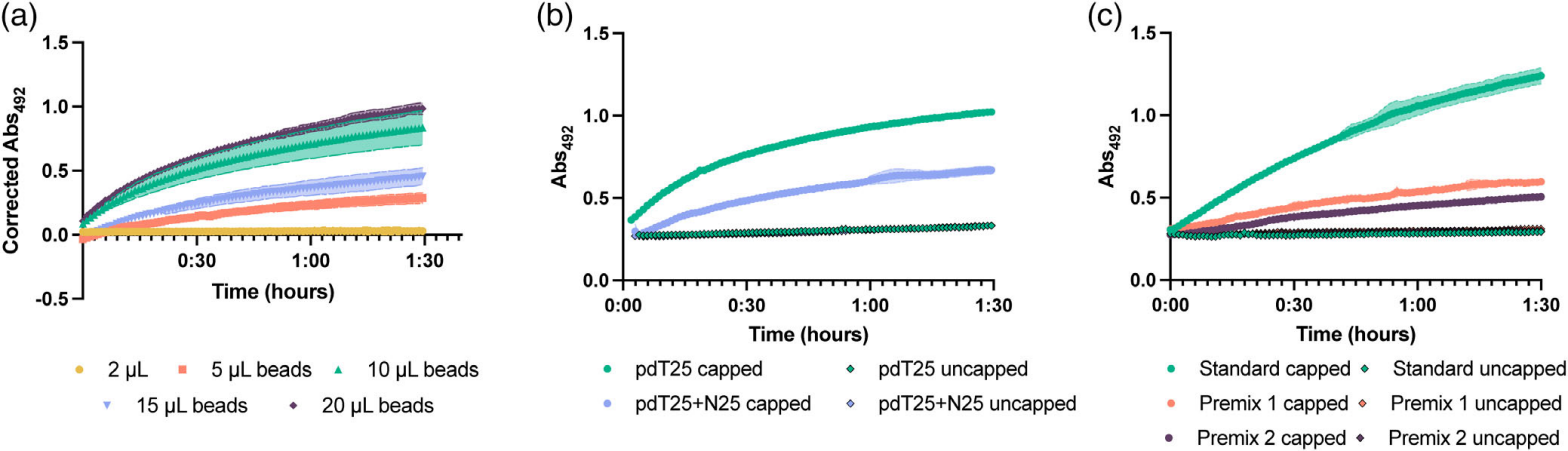
Maximizing the initial RNA capture for improved biosensor assay functionality. (a) Altering the volume of beads used in the RNA capture impacts the RNA capture. Bead slurry volumes ranging from 2 to 20 μL were used. Samples were run in triplicate (capped) and adjusted for the negative control (uncapped). (b) Increasing the length of the poly‐dT oligo using randomized DNA sequence as filler reduces the biosensor signal. Oligos of 25 nucleotides (all dT) or 50 nucleotides (25 dT plus 25 randomized nucleotides) were used. Error bars are the standard deviation of triplicate reactions of both the capped and uncapped (negative control). (c) Preincubation of the RNA with the poly‐dT oligo reduces the biosensor signal. Three conditions were run; the standard condition of pre‐binding the pdT25 to the streptavidin beads, incubating the RNA and pdT25 together under standard conditions (Premix 1), incubating the RNA and pdT25 together and omitting the resuspension step in Buffer A (Premix 2). Error bars are the standard deviation of triplicate reactions of both the capped and uncapped (negative control).

Prior to using DoE to optimize the RNA biosensor, three factors that did not fit within the framework of DSD were investigated separately to determine their impact on the assay (Figure 2). We hypothesized that the efficiency of RNA capture may be reduced due to steric hindrance on the surface of the bead and that this could limit the overall signal generation reaction affecting both the signal‐to‐noise ratio and the limit of detection. Therefore, we examined three factors related to improving RNA capture. The first was the concentration of the beads used in the initial RNA capture step. The baseline conditions used 10 μL of the Dynabeads™ MyOne™ Streptavidin T1 slurry. This concentration was both increased and decreased to observe whether RNA binding could be improved, thus altering the characteristics of the biosensor (Figure 2a). For example, using a smaller number of beads in the capture step could result in a higher density of RNA on the surface of the support and this could result in a higher signal. Alternatively, if steric hindrance on the surface of the beads due to high RNA coverage prevents downstream binding steps from occurring efficiently, then increasing the number of beads in the capture step could improve the signal by reducing RNA density. Therefore, we evaluated bead slurry volumes ranging from 2 to 20 μL in the RNA capture step. Decreasing the number of beads decreased the signal from the biosensor, with the assay containing 2 μL of bead slurry in the initial capture step showing only 30% of the activity of the baseline conditions at the end of the 90‐min reaction, whereas using 5 μL of bead slurry retained 65% of the activity. Interestingly, the assay containing 15 μL of bead slurry did not follow this linear trend and showed an 18% reduction in activity compared to the original conditions. Increasing the volume of bead slurry used in the initial capture step to 20 μL did show approximately 7% increase in activity compared to the baseline conditions, but this was not statistically significant using an unpaired t‐test (*p* = 0.143). Also, taking into consideration the cost of the beads, we decided that this increase was not enough to warrant the additional costs that would be associated with doubling the bead concentration in the capture step.

The second factor we investigated was the distance between the bead surface and the oligo dT used to capture the RNA. In the original design, a biotinylated 25 nucleotide poly‐dT oligo was used.^17^ However, we hypothesized that moving the dT further away from the surface might enhance RNA capture by giving greater flexibility and reducing steric hindrance. Therefore, a 25‐nucleotide spacer was added between the biotin and the poly‐dT sequence. The spacer was designed using a random DNA generator to ensure that no bias, secondary structure, or self‐interaction was introduced (Table S1). The two oligos were compared in a standard biosensor assay (Figure 2b). Contrary to our hypothesis that introducing a spacer may reduce steric hindrance, it is apparent that the longer oligo significantly impedes the signal of the biosensor (by ~35%). Therefore, the initial poly‐dT oligo (pdT_25_) was used for the optimization study.

Finally, the biosensor assay involves capturing the RNA via the poly‐dT oligo that was already bound to the beads. However, it is possible that the 3D conformation of the RNA prevents it from binding efficiently. Therefore, we tested whether preincubation of the poly‐dT oligo with the RNA, followed by binding the complex to the streptavidin beads would improve the biosensor characteristics. Two different preincubation experiments were performed. In both cases, 0.3 μg of RNA and 0.3 μM of poly‐dT oligo were mixed at room temperature for 30 min on a rotator at 60 rpm followed by mixing with the beads. Subsequently, the beads were washed and resuspended in 2X buffer A (Premix 1) as in the baseline assay or this step was omitted (Premix 2). Figure 2c shows that the biosensor signal is reduced under both assay conditions. It is possible that the preincubation prevents binding of the RNA‐poly‐dT oligo complex to the streptavidin beads. Omitting the step where the beads are reuspended in 2X buffer A (Premix 2), significantly further reduced the effectiveness of the biosensor (unpaired *t*‐test at end point *p* = 0.0002), further highlighting the importance of this step in the assay. Therefore, the addition of components was left as in the baseline assay (Figure 1b) for the optimization experiments.

### 3.2 Definitive screening design for an improved RNA biosensor assay

**FIGURE 3:**
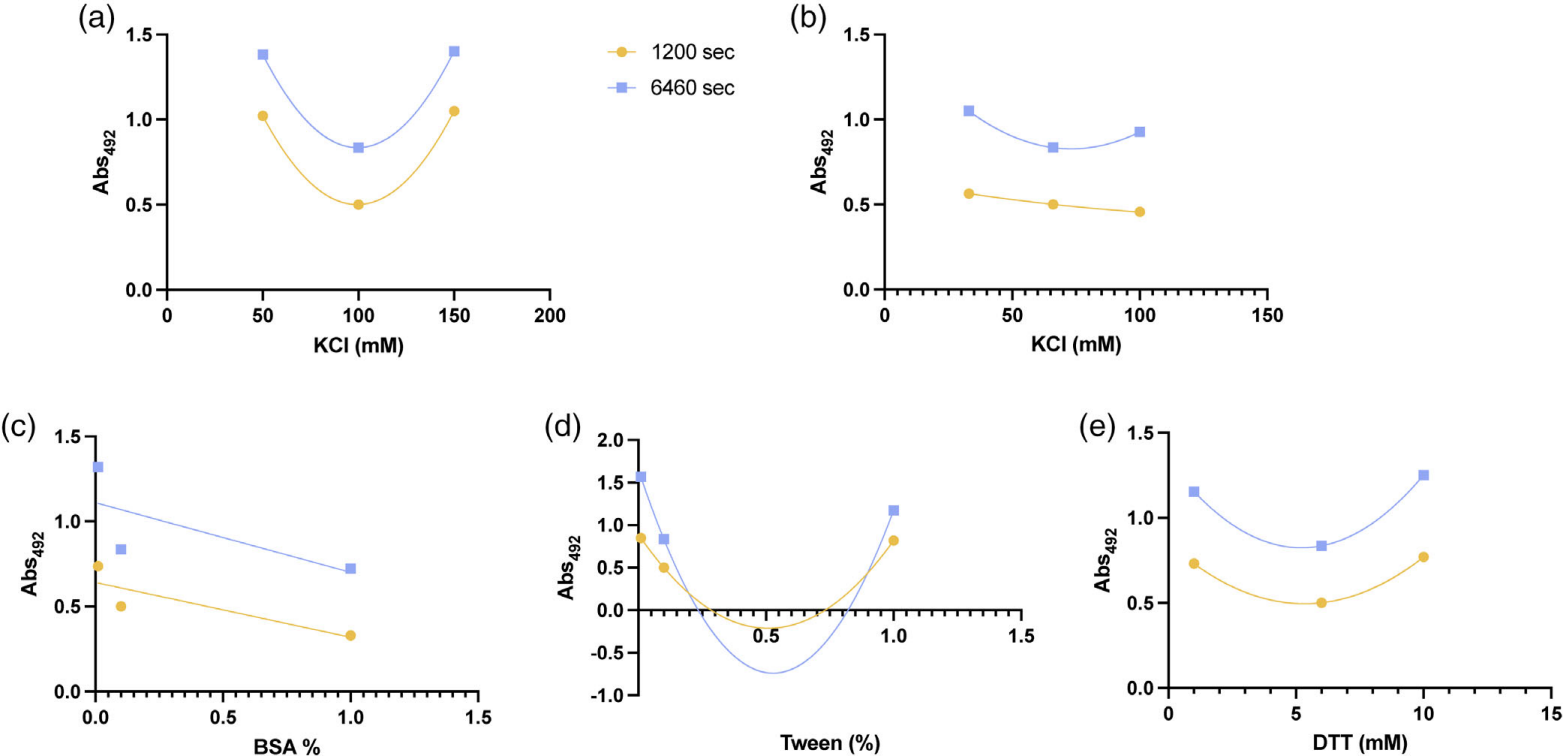
Establishing upper and lower bounds for the definitive screening design. Average Abs492 taken at either 1200 s or 6460 s of the biosensor assay plotted against the low, medium or high conditions for each factor (*n* = 3). (a) Salt concentration in RNA buffer, (b) Salt concentration in protein buffer, (c) BSA concentration (%), (d) Tween‐20 concentration (%), (e) DTT concentration (mM).

For any DoE, the design space needs to be pre‐established with upper and lower bounds chosen so that all designed runs result in a non‐zero signal. We initially selected nine factors that were thought to be important for the assay: the salt concentration and pH of the RNA buffer, the pdT_25_ concentration, the pH and concentrations of salt, BSA, Tween‐20 and DTT in the protein buffer, and the concentration of the B4E protein. Our previous work had established a range of pH of the protein and RNA buffers and B4E protein and pdT25 concentrations that could be used in the screen.^17^ However, for the remaining five conditions upper and lower bounds were tested at two different time points to identify suitable ranges (Figure 3, Figure S1). Each of the lower, mid, and upper bound conditions for the individual factors gave a non‐zero signal, with differing impacts on the assay and, as expected, there was not always a linear correlation between the factor and biosensor signal (Figure 3). Changing the KCl concentration in the protein buffer (Figure 3b) did not have notable impact on the assay, with a difference of less than 0.2 absorbance units across the three conditions, and as a result it was dropped from the DSD. For the other factors, we used the established conditions as the upper and lower bounds.

The remaining eight conditions were inputted into the JMP Pro 15 DSD program with the pre‐defined lower, mid and upper bounds (Table S2). The runs were split into three blocks with center runs included to monitor variability and four extra runs included by the JMP software to expand the design space, totaling 23 reactions (Table 1). The center runs are included as a component of the model and blocks are scaled accordingly to take into consideration any differences that occur from running experiments on separate days (Figures S2B, S2P, S2R). Each experiment included a negative control, to ensure that the changes observed were not because of an increase in the background signal irrespective of the presence of RNA (Figure S2). To decide which output to use in model fitting, we considered a variety of factors. Firstly, while we were interested in assay conditions that led to an increase in signal, as indicated previously, we did not want this to be at the expense of a high background, so fitting a model on the maximum absorbance was not sufficient. For instance, in Figure S2E, both the signal and background are high, suggesting a poor set of conditions. Therefore, we examined different transformations of the signal against the negative control, either an adjustment, where the negative control was subtracted (‘end adjusted’), or a ratio where the signal was divided by the negative control (similar to a signal‐to‐noise ratio, ‘end ratio’). We also considered whether we could identify conditions that led to a faster initial rate of reaction, by fitting the model to the output at an early time point in the reaction (*t* = 10 min, ‘mid‐adjusted’), rather than the endpoint. All models were run as forward stepwise regression models using a *p*‐value of 0.1.

**TABLE 1:** Definitive screening design of initial 23 run including center runs for three blocks and four additional runs.

Block RNA	[NaCl]	mM	RNA pH	[pdT25] μM	[Protein] μM	BSA (%)	Tween‐20 (%)	[DTT] mM	pH Protein
1	1	150	9	10	0.122	1	0.1	10	6
2	1	100	7.5	5.5	0.366	0.55	0.55	5.5	7.5
3	1	150	6	1	0.366	1	1	10	9
4	1	50	6	1	0.61	0.1	1	1	9
5	1	150	9	1	0.61	0.1	1	5.5	6
6	1	100	6	1	0.122	0.1	0.1	1	6
7	1	50	9	10	0.366	0.1	0.1	1	6
8	1	50	6	10	0.122	1	0.1	5.5	9
9	1	100	9	10	0.61	1	1	10	9
10	2	150	9	1	0.61	1	0.1	1	7.5
11	2	50	6	10	0.122	0.1	1	10	7.5
12	2	150	6	10	0.61	0.55	0.1	1	9
13	2	50	7.5	10	0.61	1	1	1	6
14	2	150	7.5	1	0.122	0.1	0.1	10	9
15	2	50	9	1	0.122	0.55	1	10	6
16	2	100	7.5	5.5	0.366	0.55	0.55	5.5	7.5
17	3	50	6	1	0.61	1	0.1	10	6
18	3	100	7.5	5.5	0.366	0.55	0.55	5.5	7.5
19	3	150	9	10	0.122	0.1	1	1	9
20	3	50	9	5.5	0.61	0.1	0.1	10	9
21	3	50	9	1	0.122	1	0.55	1	9
22	3	150	6	10	0.61	0.1	0.55	10	6
23	3	150	6	5.5	0.122	1	1	1	6

**FIGURE 4:**
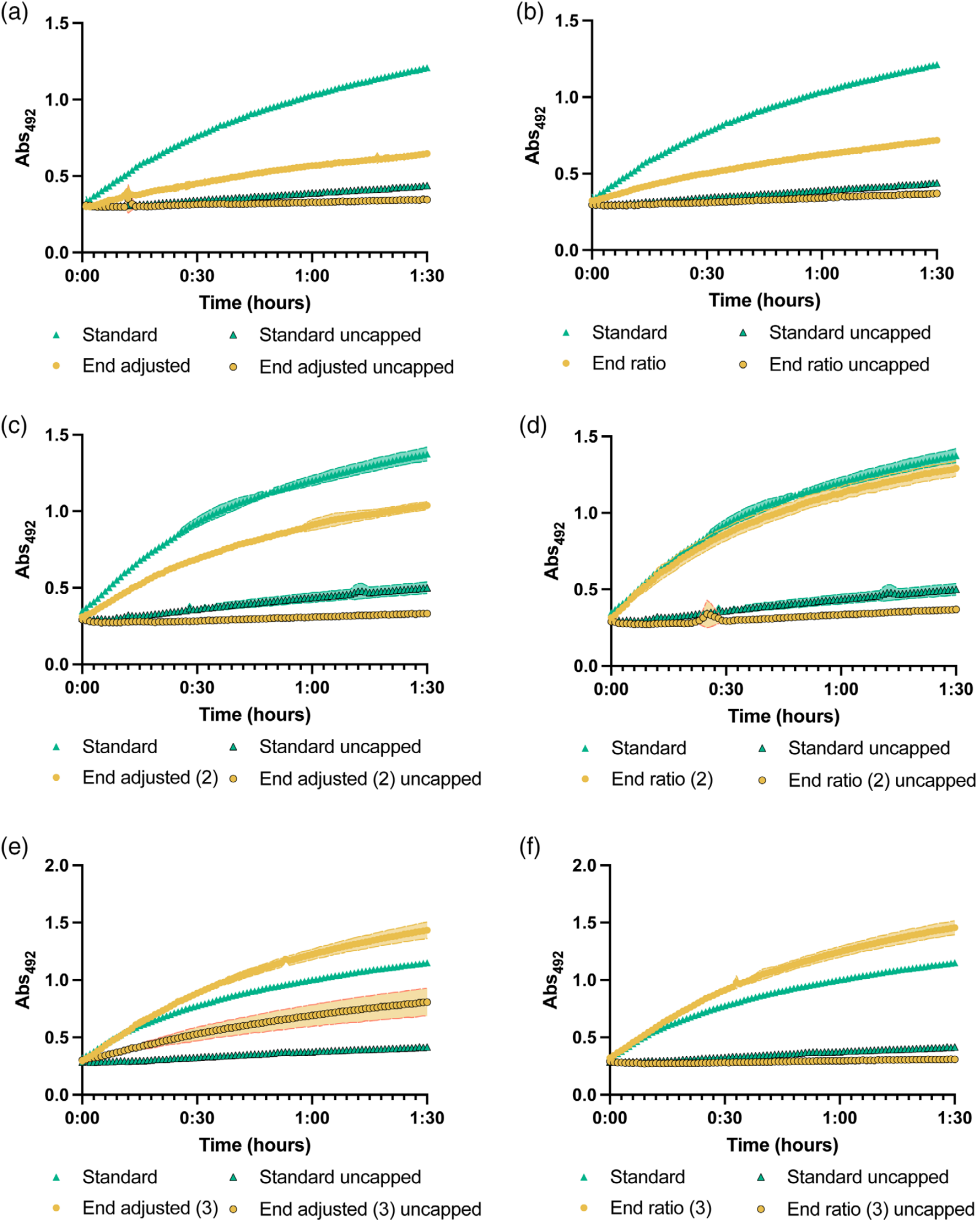
Biosensor assay response curves using conditions suggested from the definitive screening design using different model algorithms. Error bars are the standard deviation of triplicate reactions. (a) End adjusted model round 1, (b) End ratio model round 1, (c) End adjusted model round 2, (d) End ratio model round 2, (e) End adjusted model round 3, (f) End ratio model round 3.

Each of the three models identified different key factors. For instance, the ‘end adjusted’ model identified the concentrations of DTT and pdT_25_ as well as second order interactions of the protein buffer pH with itself and the concentration of DTT as the key contributing components (Figure S3). The ‘end ratio’ model also identified the concentration of pdT_25_ and the second order interaction of protein buffer pH as important, along with the protein buffer pH. Finally, the ‘mid adjusted’ model, identified a larger number of statistically significant factors, some of which overlapped with the other models (e.g., DTT and pdT_25_ concentration) and some of which did not (e.g., RNA buffer pH, RNA buffer salt concentration, second order interaction of RNA salt concentration and protein concentration, for a full list see Figure S4A). To determine which model works best toward the goal of improving the RNA biosensor, each was used to predict conditions that maximize the ‘end ratio’, ‘end adjusted’ or ‘mid adjusted’ values, respectively, (Table S3), and a set of reactions corresponding to these conditions was run (Figure 4a,b, Figure S4B). For each, a run with the baseline conditions^17^ was included, as well as surplus runs identified by alternative ways of fitting the model to provide extra data for refinement (e.g., using standard fitting instead of a stepwise regression).

What is immediately noticeable is that using the conditions indicated by the ‘mid adjusted’ model improved the speed of the response of the biosensor, but also increased the background signal of the negative control (Figure S4B). Therefore, this is not a suitable model for the improvement of the RNA biosensor and it was removed from all future analyses. The conditions suggested by both the ‘end adjusted’ and ‘end ratio’ models resulted in a decrease in the absolute absorbance compared to the original conditions; however, the negative control was also reduced (Figure 4a,b), leading to better signal‐to‐noise ratio.

Next, both the ‘end adjusted’ and ‘end ratio’ models were run again incorporating the additional data from the optimized experiments (Table S4), totaling 29 runs including an additional center run (Figure S3C,D). For both models the most important factor was the two‐factor interaction of protein concentration with itself. While there were other similarities, such as the significance of the interaction between protein buffer pH with itself, and the two‐factor interaction between pdT25 and Tween, there were some differences as well, such as the concentration of BSA being significant in the ‘end ratio’ model but not in the ‘end adjusted’ model. Additionally, the number of components identified as statistically significant by the ‘end ratio’ model was higher (12 vs. 10). Once again, both models were used to suggest optimized conditions (Figure 4c,d). While neither set of conditions showed an improvement in the signal of the biosensor compared to the baseline conditions, both showed a decrease in the absolute absorbance of the negative control, leading to further improvement in the signal‐to‐noise ratio (2.74 for the baseline conditions, 3.11 for the conditions suggested by the ‘end adjusted’ model, and 3.51 for the conditions suggested by the ‘end ratio’ model). The reduction of the background of the assay led to an improvement in the dynamic range of the biosensor showing both models are suitable for exploring further optimizations.

Due to the improvements observed in the second round, a third round of optimization was performed, again using the data generated previously, as well as incorporating the data from round 2 from the conditions suggested by both models (Table S5). This resulted in an additional 5 conditions totaling 34 runs, set over 5 blocks with 5 center points included (Figure S3E,F). Similar factors had a significant impact on the model as in previous iterations, such as the two‐factor interaction of protein concentration with itself, but whereas previously both models showed the interaction between pdT_25_ and Tween to be significant, only the ‘end ratio’ model shows that this is a significant component in round 3.

The conditions suggested by both models result in an improvement in the absolute absorbance of the RNA biosensor (Figure 4e,f). For the conditions suggested from the ‘end adjusted’ model, an endpoint absorbance of 1.44 was observed, compared to 1.15 from the baseline conditions. This was similar to the conditions suggested by the ‘end ratio’ model, which gave an endpoint absorbance of 1.46. Both showed an increased signal of approximately 25% compared to the baseline conditions. However, the ‘end adjusted’ model also showed an increase in the baseline of the negative control, meaning the signal‐to‐noise ratio was reduced from 2.77 to 1.77. Comparatively, the negative control from the conditions suggested by the ‘end ratio’ model maintained the reduced signal as seen from the second round of optimization, resulting in a signal‐to‐noise ratio of 4.71. This is an increase of 1.7‐fold compared to the baseline conditions. This suggests the ‘end ratio’ model better captures the interplay between the signal and the background.

To determine whether the conditions (Table S6) had converged, the ‘end ratio’ model was once again run, populated with the data from the previous optimizations, with a total of 39 runs across 6 blocks with 6 center points. There was near convergence with the previous conditions (Table S5); the only difference was a reduction in protein concentration from 0.45 to 0.2 μM (Figure S5). This provided confidence that the conditions that we had identified were a local optimum for maximizing both the signal and the signal‐to‐noise ratio of the RNA biosensor.

### 3.3 Impact of the new design on RNA sample concentration requirements

**FIGURE 5:**
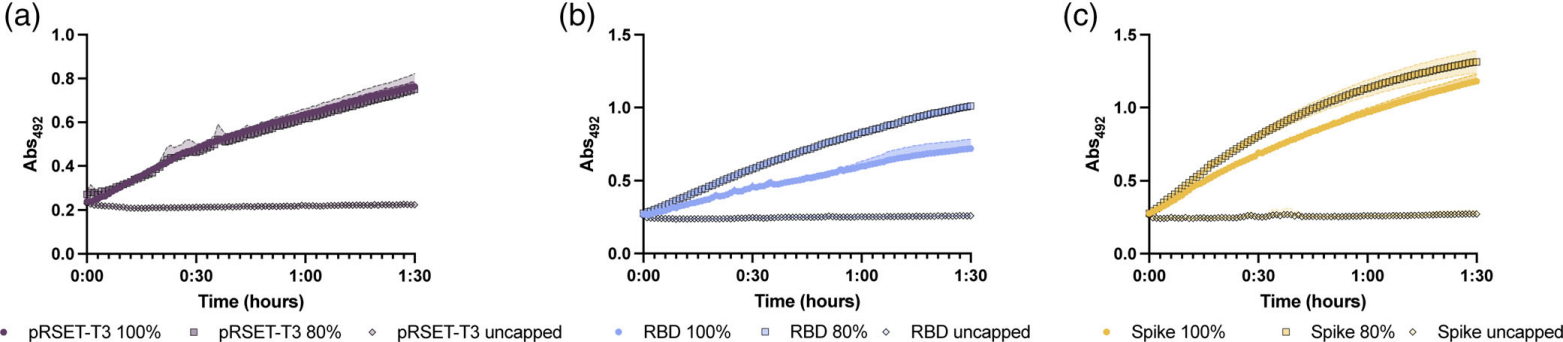
Biosensor tests using different concentrations of capped RNA of different length. (a) pRSET‐T3 RNA (2700 nt, (b) CFPS‐RBD RNA (700 nt), (c) CFPS‐Spike RNA (3800 nt). Error bars are the standard deviation of triplicate reactions.

To test the functionality of the biosensor, we mixed different ratios of capped and uncapped RNA to observe whether the biosensor is capable of discerning differences in the quality of the RNA (Figure 5a). Furthermore, we were interested in observing how the limit of detection changes with different length RNAs, as we previously observed that the signal was reduced with longer RNA sequences.^17^ Therefore, in addition to using the pRSET‐T3 linearised template, we also used two additional templates, a CFPS‐RBD template (IRES‐mediated expression of the receptor binding domain from SARS‐CoV2), and a CFPS‐Spike template (IRES‐mediated expression of the spike protein), which produce RNA of 700 nt and 3800 nt, respectively (Figure 5). Our optimized conditions resulted in an inability of the biosensor to accurately distinguish between RNA that was 100% capped and that which was only 80% capped. For the pRSET‐T3 target, there was no significant difference in signal between the two. More interestingly, both CFPS‐RBD and CFPS‐Spike RNA showed a higher signal for samples with 80% capped RNA compared to those with 100% capped RNA. This inversion suggested to us that the signal might now be oversaturated and that reducing the concentration of the RNA, which would ultimately be beneficial for any screening assay, especially with long RNAs such as vaccines, could improve the system.

**FIGURE 6:**
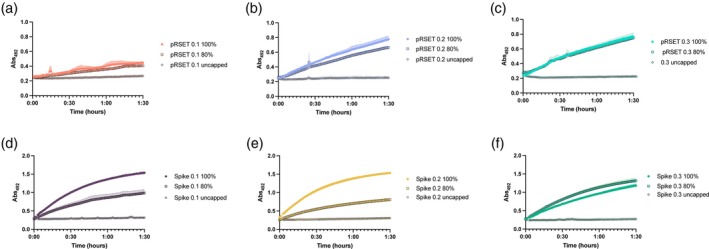
Reducing RNA concentrations improves the functionality of the biosensor assay with the optimized conditions. (a) pRSET 0.1 μM RNA, (b) pRSET 0.2 μM RNA, (c) pRSET 0.3 μM RNA, (d) Spike 0.1 μM RNA, (e) Spike 0.2 μM RNA, (f) Spike 0.3 μM RNA. Error bars are the standard deviation of triplicate reactions.

To test our hypothesis that lowering the concentration of RNA used in the biosensor assay would restore its ability to discriminate between samples with larger amounts of capped RNA, samples containing pRSET‐T3 and CFPS‐Spike RNA were run using total concentrations of 0.3, 0.2, and 0.1 μM with 80% or 100% capping (Figure 6). Both the lower concentrations of RNA restored the ability of the biosensor to be able to distinguish between 100% capped and 80% capped RNA. However, for the 0.1 μM total concentration of pRSET‐T3 RNA, the final absorbance signal was reduced, and therefore we propose using 0.2 μM RNA for all future assays.

## 4 DISCUSSION

Design of experiments allowed us to evaluate a large design space for the improvements of the biosensor assay to assess RNA capping and integrity. One of the most interesting aspects was how robust the original conditions were, with multiple iterations of the DoE model required before the conditions outcompeted the original conditions in both signal‐to‐noise ratio and limit of detection. Notable modifications were the decrease in both the B4E protein and the pdT_25_ concentrations, as well as an increase in the amount of DTT required, suggesting a reducing environment as the best condition for full functionality. The decrease in pdT_25_ concentration may be partly responsible for the oversaturation behavior at high RNA concentrations under the optimized conditions. In addition, self‐oligomerization of B4E through disulfide bond formation has previously been observed during purification,^26^ which is potentially being prevented through the increased concentration of DTT.

The improvement of the biosensor conditions resulted in a reduction in the amount of RNA needed for the assay. This unexpected effect will ultimately be beneficial especially when dealing with longer RNA, such as the Covid19 mRNA vaccines which were 4284 nucleotides in length.^27^ The difference has a substantial impact, a reduction by a third of the usage of RNA, irrespective of length, will reduce the amount of material required for testing, potentially increasing the feasibility of using this test for manufacturing process optimization, high throughput screening, or in the field without concerns about depleting supply.

The use of nitrocefin in the assay can result in high backgrounds due to the high concentration of the substrate added, which will reduce the sensitivity of the assay.^28^ Switching to a fluorescent substrate, such as CCF2‐FA, which can be used at a lower concentration could further increase the sensitivity of the assay leading to a further reduction in the amount of RNA required. The advantage of using DSD over alternative DoE methods is the reduced number of runs required to achieve an optimized biosensor.^18^ This applicable method will allow easy optimization in the event of further modifications to the biosensor assay, for instance if an alternative fluorescent substrate is used. Furthermore, our approach of taking into consideration both the total absorbance and the background shows the power of design of experiments, as a one‐factor at a time approach may not have yielded such an overall improvement in the assay, particularly where interactions between factors are identified. Our proposed workflow incorporating DSD and experimental validation would be applicable to other biosensor designs with quantitative outputs, suggesting the versatility of our process. Ultimately, our biosensor now not only has both improved characteristics but, also a reduced amount of RNA required for use.

## AUTHOR CONTRIBUTIONS

**Rochelle Aw:** Conceptualization; investigation; writing – original draft; formal analysis; methodology; validation. **Karen Polizzi:** Conceptualization; funding acquisition; writing – review and editing; project administration; supervision.

## CONFLICT OF INTEREST STATEMENT

The authors have no conflict of interest to declare.

## Supporting information


**Data S1**.

## Data Availability

The data that support the findings of this study are available from the corresponding author upon reasonable request.
