# Supplementary material for: Enhancing the performance of an in vitro RNA biosensor through iterative design of experiments
Source: Biotechnol Prog. 2025 Mar 12;41(4):e70005. doi: 10.1002/btpr.70005 (PMC12348311; doi:10.1002/btpr.70005)
Supplement: Supplementary file 1 — Data S1. [file BTPR-41-e70005-s001.docx]

Enhancing the performance of an *in* vitro RNA biosensor through iterative design of experiments

Rochelle Aw^1,2,3^ and Karen Polizzi^1,2, *^

^1^Centre for Synthetic Biology, Imperial College London, London, SW7 2AZ, UK

^2^Department of Chemical Engineering, Imperial College London, London SW7 2AZ, UK

^3^Current address: Department of Bioengineering, Stanford University, Stanford, CA, USA

*To Whom Correspondence Should Be Addressed: [k.polizzi@imperial.ac.uk](mailto:k.polizzi@imperial.ac.uk)

**Supplementary Figures**


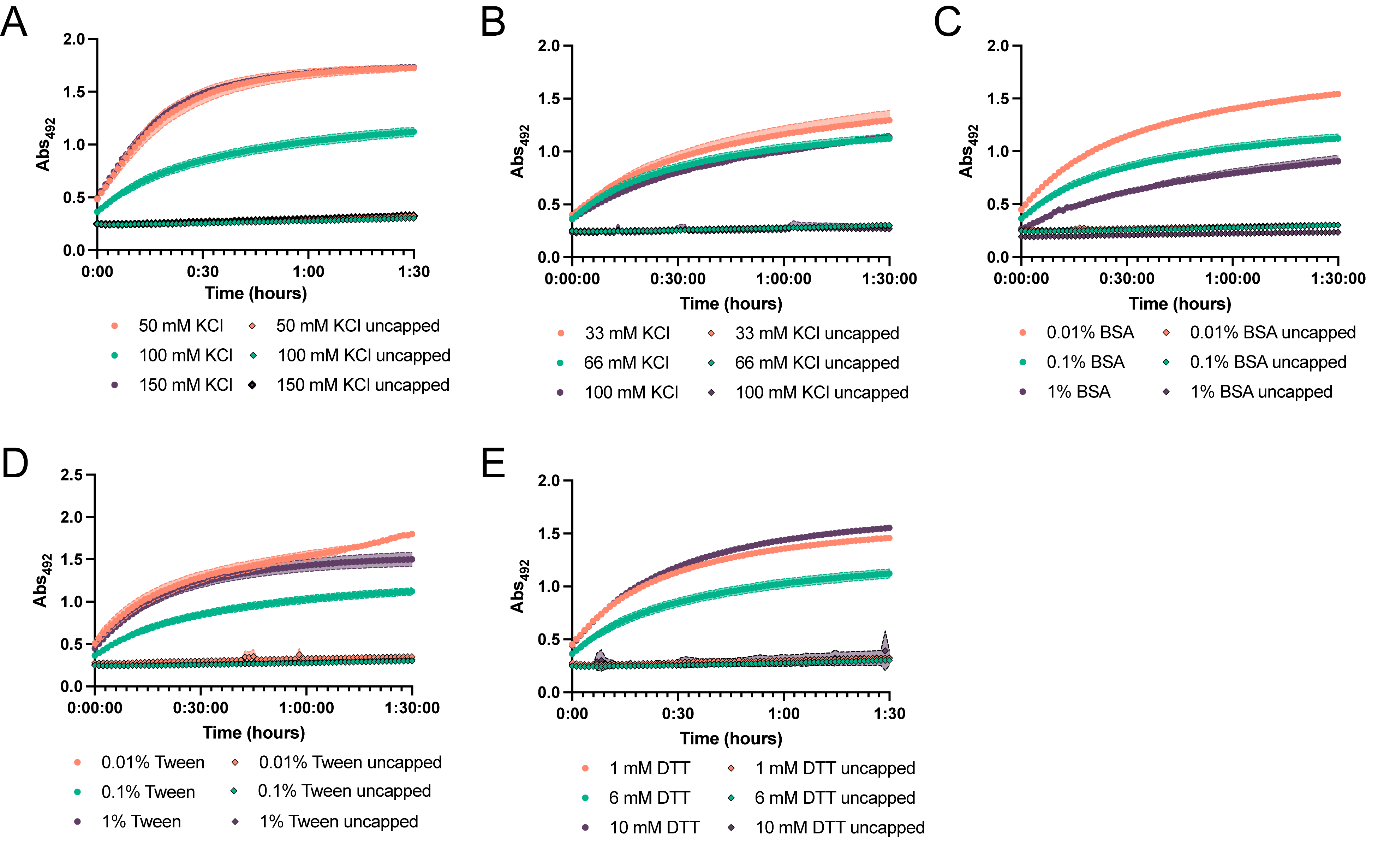


**Supplementary Figure 1.** Biosensor assay responses to different concentrations setting the upper and lower bounds for the definitive screening design. Error bars are the standard deviation of triplicate reactions. A) Salt concentration of RNA buffer, B) Salt concentration of protein buffer, C) BSA concentration (%), D) Tween-20 concentration (%), E) DTT concentration (mM).


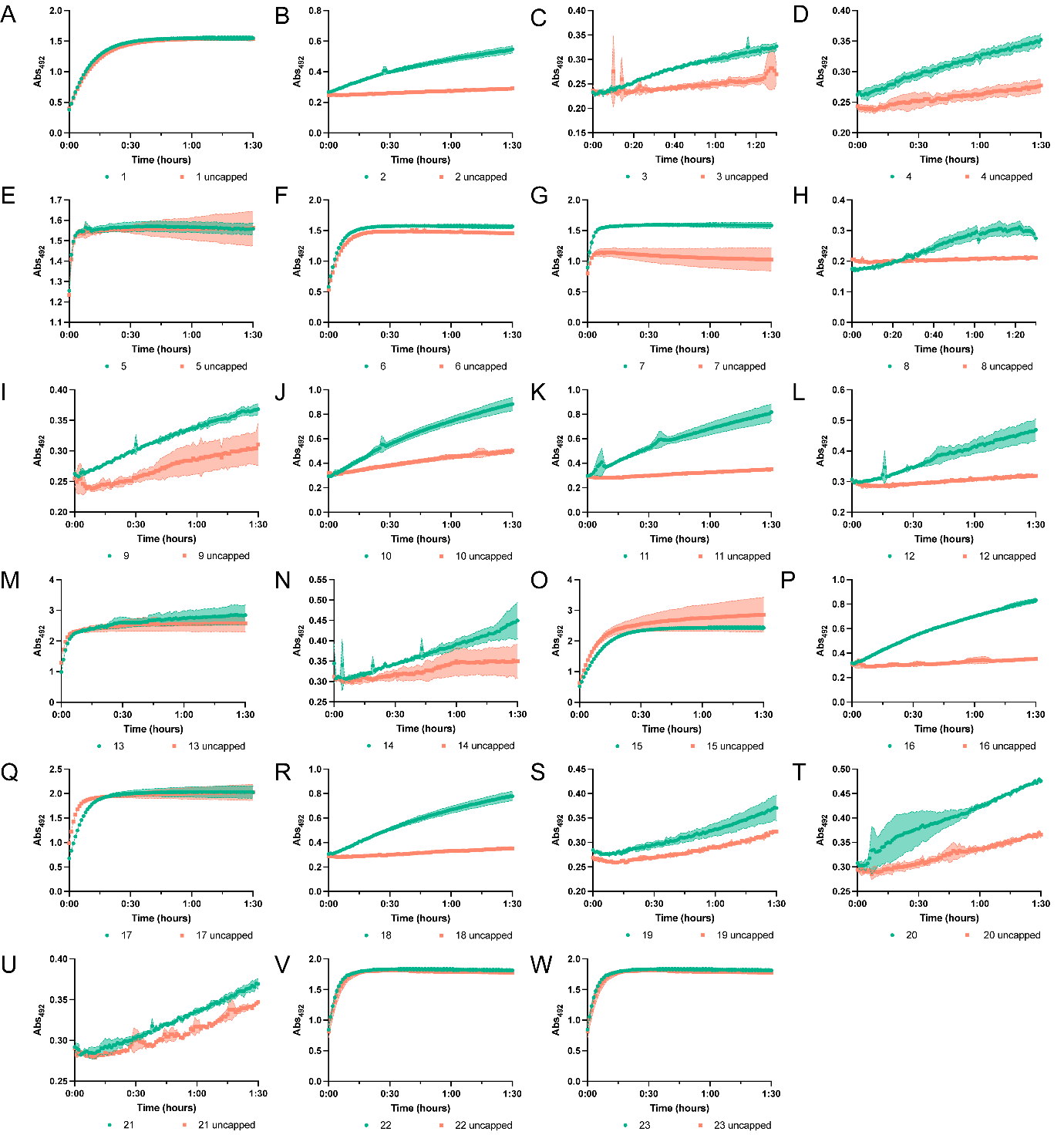


**Supplementary Figure 2.** Biosensor assay responses to the initial 23 runs using the conditions outlined in Table 1. Runs 1-27 are represented in panels A-W respectively. Error bars are the standard deviation of triplicate reactions.


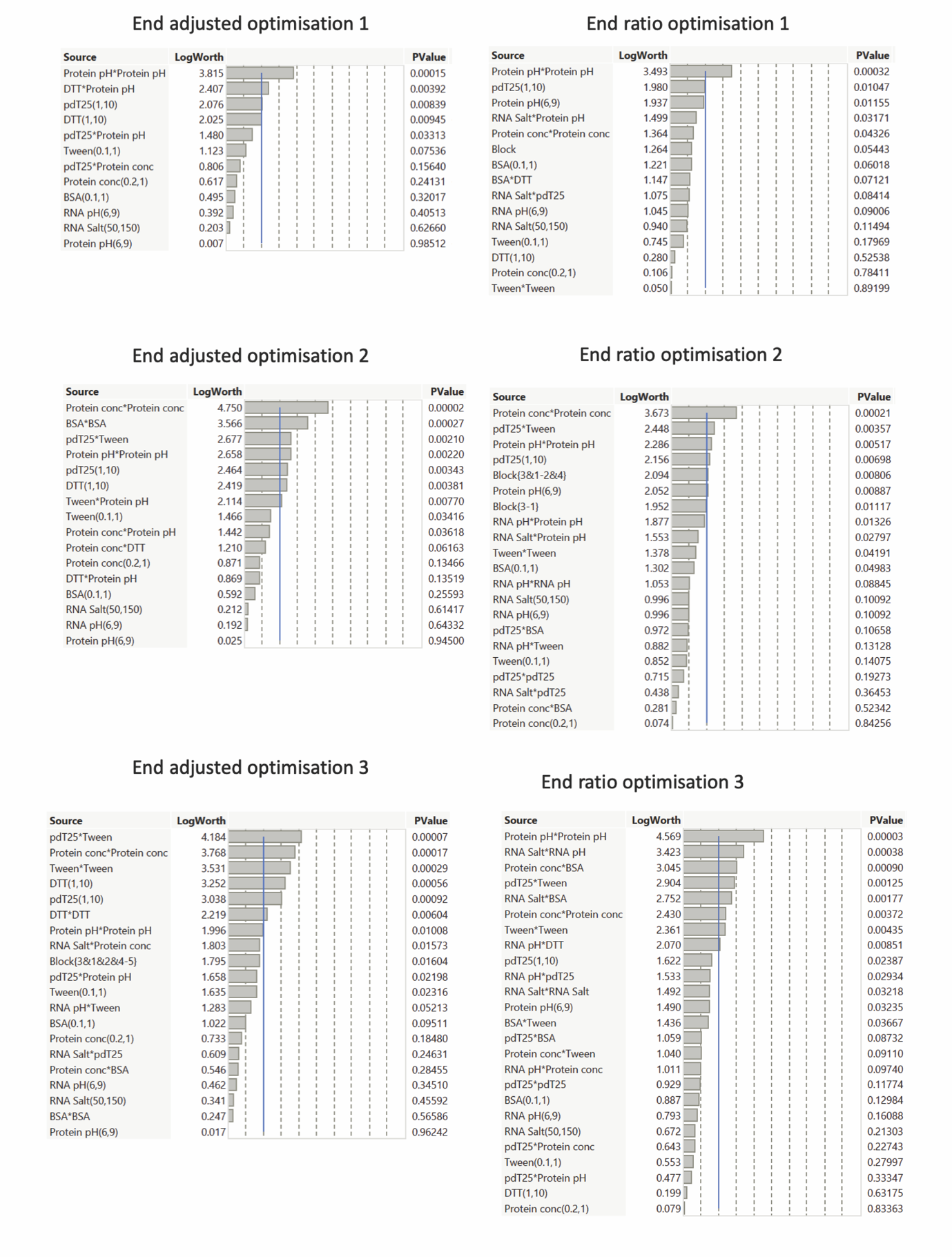


**Supplementary Figure 3.** Key components of each model as predicted by JMP software for various iterations of runs and model conditions, either using the end adjusted data set or the end ratio dataset.


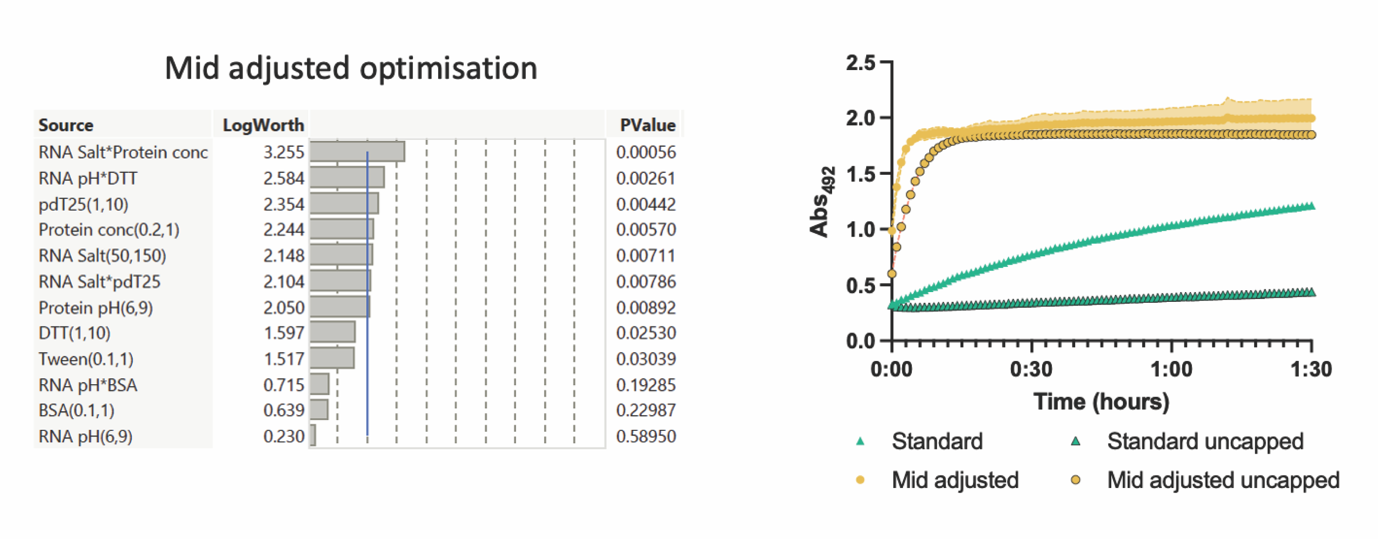


**Supplementary Figure 4.** Model output using ’mid-adjusted’ data set. A) Key components are predicted by JMP software. B) RNA biosensor response curve with the conditions as suggested from the maximized desirability settings. Error bars are the standard deviation of triplicate reactions.


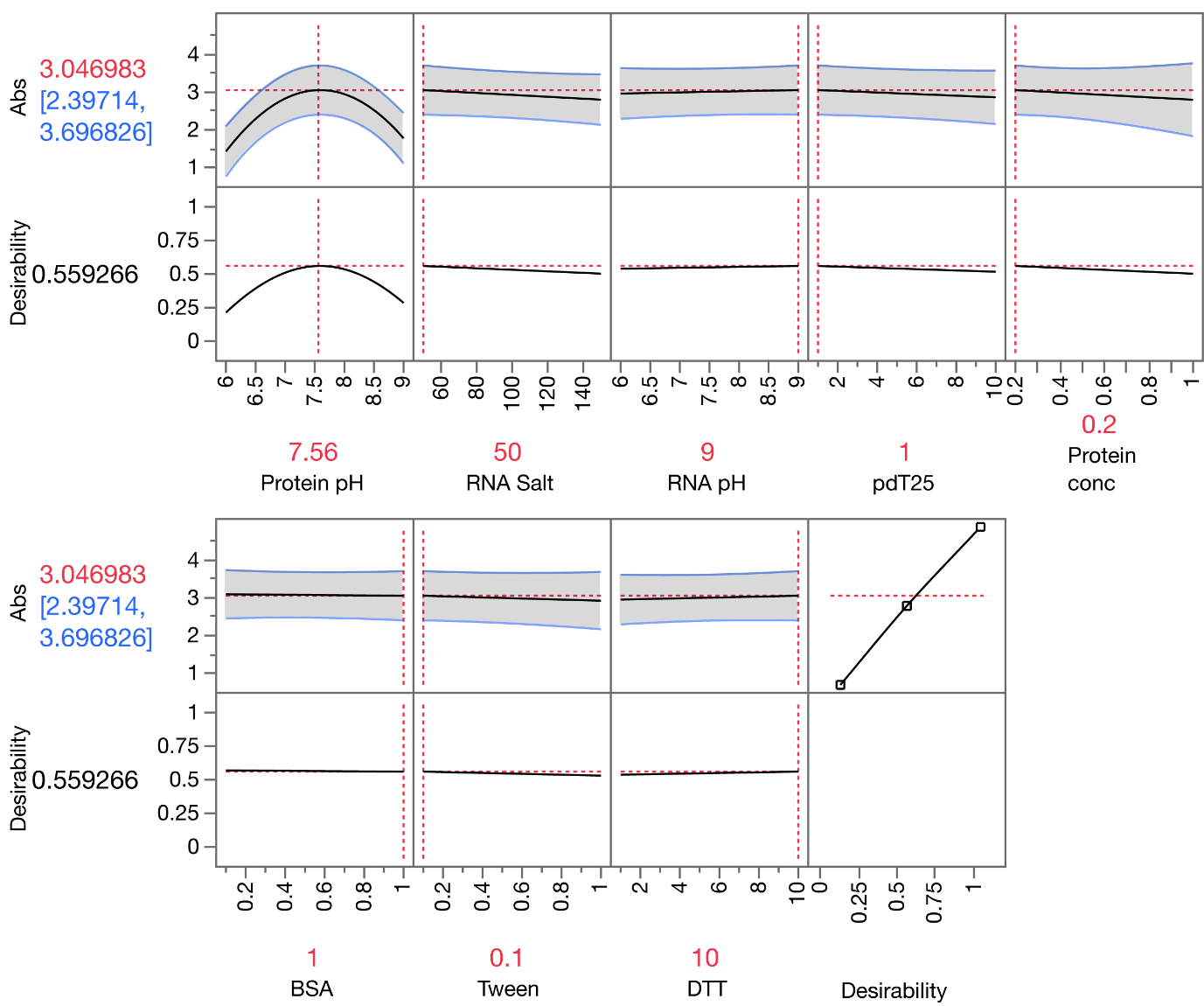


**Supplementary Figure 5.** Final model output from maximized desirability after four rounds of a definitive screening design using the end ratio model.

**Supplementary Tables**

Table S1. DNA sequence of the pT25+25N primer

| Primer | Sequence |
| --- | --- |
| pdT_25_+25N | TTTTTTTTTTTTTTTTTTTTTTTTTGTTATGATCTCTTACATTTTACTAT |

Table S2. Lower, mid and upper bounds for the eight conditions used in the DSD

|  | Lower | Mid | Upper |
| --- | --- | --- | --- |
| RNA [NaCl] mM | 50 | 100 | 150 |
| RNA pH | 6 | 7.5 | 9 |
| [pdT25] µM | 1 | 5.5 | 10 |
| [Protein] µM | 0.122 | 0.366 | 0.61 |
| BSA (%) | 0.1 | 0.55 | 1 |
| Tween-20 (%) | 0.1 | 0.55 | 1 |
| [DTT] mM | 1 | 5.5 | 10 |
| pH Protein | 6 | 7.5 | 9 |

Table S3. Suggested maximum desirability conditions after one round of DSD using different model parameters

|  | RNA Salt | RNA pH | pdT25 | Protein conc | BSA | Tween | DTT | Protein pH |
| --- | --- | --- | --- | --- | --- | --- | --- | --- |
| End adjusted | 50 | 6 | 10 | 0.2 | 0.1 | 0.1 | 1 | 7.5 |
| End ratio | 50 | 6 | 6.85 | 0.2 | 0.1 | 0.1 | 1 | 7.5 |
| Mid adjusted | 50 | 9 | 10 | 1 | 1 | 0.1 | 1 | 6 |
| Original conditions | 100 | 7.4 | 3.75 | 0.45 | 0.1 | 0.1 | 6 | 7.4 |

Table S4. Suggested maximum desirability conditions from the second round of DSD using different model parameters

|  | RNA Salt | RNA pH | pdT25 | Protein conc | BSA | Tween | DTT | Protein pH |
| --- | --- | --- | --- | --- | --- | --- | --- | --- |
| End Adjusted 2 | 100 | 7.5 | 10 | 0.63 | 0.1 | 1 | 1 | 7.34 |
| End Ratio 2 | 50 | 6 | 1 | 0.61 | 0.1 | 0.1 | 1 | 7.92 |
| Original condition | 100 | 7.4 | 3.75 | 0.45 | 0.1 | 0.1 | 6 | 7.4 |

Table S5. Suggested maximum desirability conditions from the third round of DSD using model parameters

|  | RNA Salt | RNA pH | pdT25 | Protein conc | BSA | Tween | DTT | Protein pH |
| --- | --- | --- | --- | --- | --- | --- | --- | --- |
| End Adustedj 3 | 150 | 6 | 10 | 0.69 | 0.1 | 1 | 4.56 | 7.11 |
| End Ratio 3 | 50 | 9 | 1 | 0.45 | 1 | 0.1 | 10 | 7.56 |
| Original condition | 100 | 7.4 | 3.75 | 0.45 | 0.1 | 0.1 | 6 | 7.4 |

Table S6. Suggested maximum desirability from the fourth and final round of SDS using end ratio model parameters

|  | RNA Salt | RNA pH | pdT25 | Protein conc | BSA | Tween | DTT | Protein pH |
| --- | --- | --- | --- | --- | --- | --- | --- | --- |
| End Ratio 4 | 50 | 9 | 1 | 0.2 | 1 | 0.1 | 10 | 7.56 |
